# Potential role of CT-textural features for differentiation between viral interstitial pneumonias, *pneumocystis jirovecii* pneumonia and diffuse alveolar hemorrhage in early stages of disease: a proof of principle

**DOI:** 10.1186/s12880-019-0338-0

**Published:** 2019-05-21

**Authors:** Christopher Kloth, Wolfgang Maximilian Thaiss, Robert Beck, Michael Haap, Jan Fritz, Meinrad Beer, Marius Horger

**Affiliations:** 1 Department of Diagnostic and Interventional Radiology, University Hospital Ulm, Albert-Einstein-Allee 23, 89081 Ulm, Germany; 2 Department of Diagnostic and Interventional Radiology, Eberhard-Karls-University, Hoppe-Seyler-Str.3, 72076 Tübingen, Germany; 3 Institute of Medical Virology, Eberhard-Karls-University, Elfriede-Aulhorn-Str. 6, 72076 Tübingen, Germany; 4 Department of Internal Medicine IV, Medical Intensive Care Unit, University of Tübingen, Otfried Müller Str. 10, 72076 Tübingen, Germany; 5 Russell H. Morgan, Department of Radiology and Radiological Science, Johns Hopkins University School of Medicine, 601 N. Caroline Street, JHOC, 3140A, Baltimore, MD 21287 USA

**Keywords:** HRCT, Pneumonia, Texture analysis, *Pneumocystis jirovecii* pneumonia

## Abstract

**Background:**

Pulmonary involvement is common in several infectious and non-infectious diagnostic settings. Imaging findings consistently overlap and are therefore difficult to differentiate by chest-CT. The aim of this study was to evaluate the role of CT-textural features(CTTA) for discrimination between atypical viral (respiratory-syncitial-virus(RSV) and herpes-simplex-1-virus (HSV1)), fungal (pneumocystis-jirovecii-pneumonia(PJP)) interstitial pneumonias and alveolar hemorrhage.

**Methods:**

By retrospective single-centre analysis we identified 46 consecutive patients (29 m) with RSV(*n* = 5), HSV1(*n* = 6), PJP(*n* = 21) and lung hemorrhage(*n* = 14) who underwent unenhanced chest CTs in early stages of the disease between 01/2016 and 02/2017. All cases were confirmed by microbiologic direct analysis of bronchial lavage. On chest-CT-scans, the presence of imaging features like ground-glass opacity(GGO), crazy-paving, air-space consolidation, reticulation, bronchial wall thickening and centrilobular nodules were described. A representative large area was chosen in both lungs and used for CTTA-parameters (included heterogeneity, intensity, average, deviation, skewness).

**Results:**

Discriminatory CTTA-features were found between alveolar hemorrhage and PJP consisting of differences in mean heterogeneity(*p* < 0.015) and uniformity of skewness(*p* < 0.006). There was no difference between CT-textural features of diffuse alveolar hemorrhage and viral pneumonia or PJP and viral pneumonia. Visual HRCT-assessment yielded great overlap of imaging findings with predominance of GGO for PJP and airspace consolidation for pneumonia/alveolar hemorrhage. Significant correlations between HRCT-based imaging findings and CT-textural features were found for all three disease groups.

**Conclusion:**

CT-textural features showed significant differences in mean heterogeneity and uniformity of skewness. HRCT-based imaging findings correlated with certain CT-textural features showing that the latter have the potential to characterize structural properties of lung parenchyma and related abnormalities.

**Electronic supplementary material:**

The online version of this article (10.1186/s12880-019-0338-0) contains supplementary material, which is available to authorized users.

## Background

Pulmonary involvement is common in several infectious and non-infectious diagnostic settings. In particular it is a diagnostic challenge in the clinical setting after bone marrow transplantation during the period of neutropenia [1]. Among them, viral and fungal pneumonias as well as diffuse alveolar hemorrhage are the most frequent and deserve prompt and adequate treatment [2–4]. Chest-CT is the most commonly involved imaging technique for detection and potential characterization of these pulmonary complications. However, imaging findings in both viral pneumonias (e.g. respiratory syncitial virus, herpes simplex-1 virus, etc.) and *Pneumocystis jirovecii* as well as in diffuse alveolar hemorrhage consistently overlap and are therefore difficult to differentiate [5–7]. Earlier reports have tried to highlight some predominant features, but there are in particular the early stages of these disorders that exhibit great similarities [3]. Bronchoscopy with bronchial lavage (BAL) is the next step after imaging using PCR-tests for PJP and virus culture or only bronchial secretion analysis for confirmation of hemorrhage and exclusion of potential pathogens [8]. Due to increased risk for hemorrhage, BAL is not always performed. For this reason, any diagnostic technique capable of making this differentiation at an early time point in the course of these pulmonary complications would be desirable. CTTA is a new technique enabling tissue characterisation in terms of structure, microarchitecture, symmetry and uniformity or heterogeneity, respectively [9]. Therefore, 1st and 2nd order statistical features are employed showing good results even in the lungs [10, 11]. The lung parenchyma has a regular, well-predictable spatial arrangement which in case of pathologic changes is expected to be more or less disturbed in a way that is reflected by the underlying pathology. Early findings in viral and PJ-pneumonias as well as in alveolar hemorrhages are confined to both the lung interstitium and the alveolar spaces.

In this project, we have aimed at potential early differentiation of viral (RSV and HSV1), PJ-pneumonias and alveolar hemorrhages based on CT-textural features.

## Methods

The local ethics board approved this retrospective study and waived informed patient consent (Study Nr.180/2017BO2).

### Study population

**Fig. 1:**
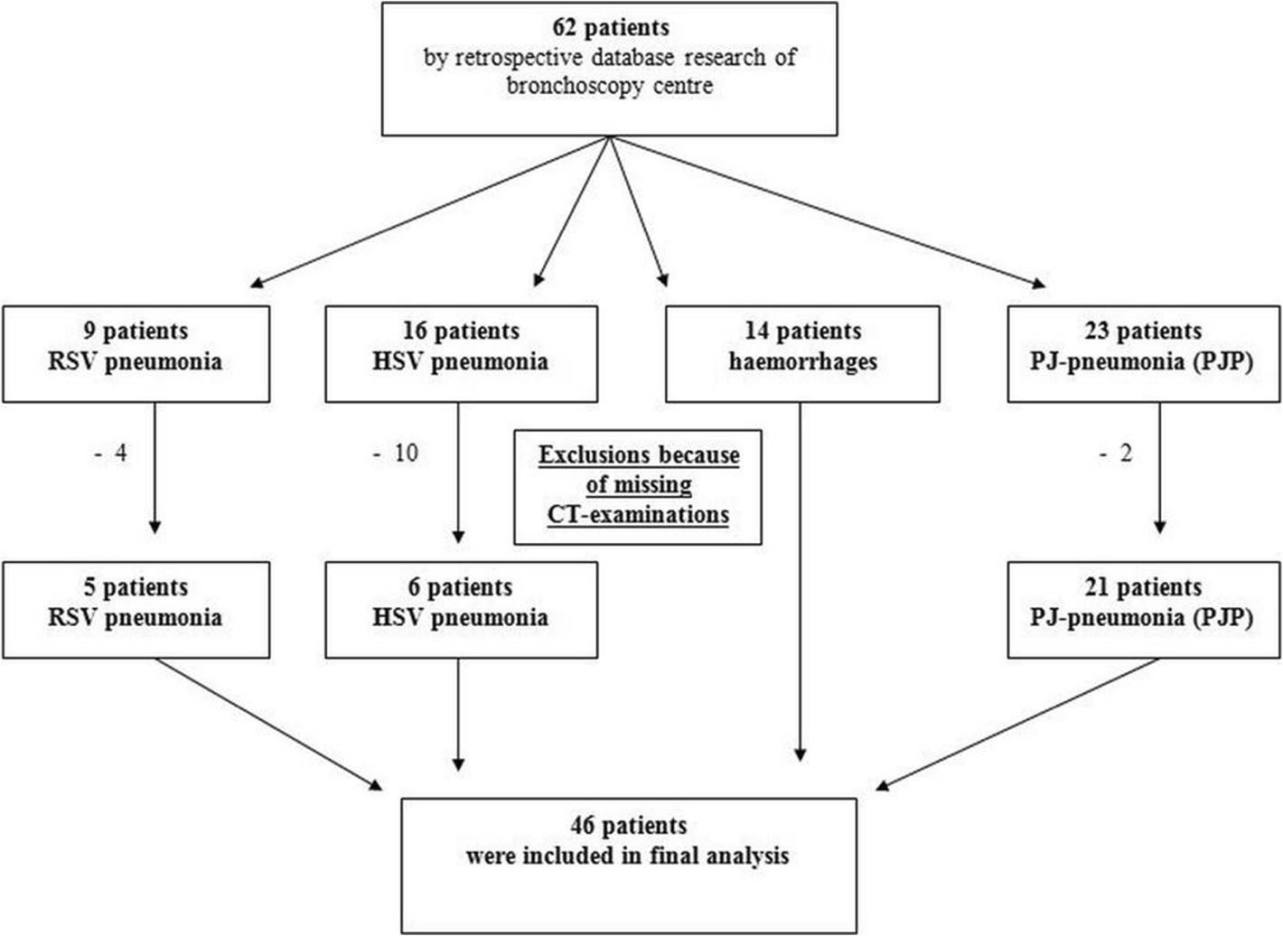
Algorithm of patient recruitment

This was a retrospective CT, clinical (microbiological) and BAL-data evaluation which was approved by the local ethic committee. By retrospective database search of the local radiology department and bronchoscopy centre we identified 62 suitable patients. Because of missing CT examinations we must exclude 16 patients, so that finally 46 patients were included (Fig. 1). These 46 consecutive patients (female, 17; male, 29; mean age 62.70y ± 14.02 y; range, 29–85 y) with RSV (*n* = 5), HSV1 (*n* = 6), PJP (*n* = 21) and lung hemorrhage (*n* = 14) underwent high-resolution chest CT within **10** days after institution of respiratory symptoms between January 2016 and February 2017. Underlying diseases and patient characteristics were shown on Table 1.

**Table 1:** Patient characteristics

Patient characteristics		
Median age (range)	62.70y ± 14.02 (29-85y)	
Sex, male/female	29/17	
	*N*	*(%)*
Underlying disease	*N* = 46	100%
*Anticoagulation*	7	15.2%
*Pneumonia*	3	6.5%
*HIV*	2	4.3%
*Fibrosis, COLD*	3	6.5%
Malignant diseases	7	15.2%
*NSCLC*	*6*	13.0%
*Melanoma*	1	2.1%
Hematologic diseases	20	43.7%
*Multiple myeloma*	4	8.6%
*Acute myeloid leukemia*	6	13.0%
*Acute lymphoblastic leukemia*	3	6.5%
*CLL*	3	6.5%
*DLBCL*	2	4.3%
*CML*	1	2.1%
*Aplastic anemia*	1	2.1%
Autoimmune diseases	4	8.6%
*Rheumatoid arthritis*	1	2.1%
*Dermatomyositis*	1	2.1%
*Systemic lupus erythematodes*	1	2.1%
*Systemic sclerosis*	1	2.1%

*Abbreviations: HIV* human immunodeficiency virus, *COLD* chronic obstructive lung disease, *CLL* chronic lymphocytic leukemia, *CML* chronic myeloid leukemia, *DLBC* diffuse large B-cell lymphoma, *NSCLC* non-small cell lung carcinoma

Mean time between bronchial lavage and CT imaging examination was 11.18 ± 1.61 days.

Patients were retrospectively recruited for both HRCT- and CTTA-analysis if they fulfilled the following inclusion criteria: 1) positive bronchoscopy for pulmonary haemorrhages, viral pneumonia or *Pneumocystis jirovecii* pneumonia; 2) at least one HRCT of the lung at the onset of the disease; 3) age over 18 years. 16 patients had to be excluded as they had no CT-diagnosis. Exclusion criteria were additional pathologies affecting the lung parenchyma and overlying the proposed clinical pathologies: 1) pleural effusions; 2) lung edema or; 3) additional bacterial infections. The process of patient recruitment is shown on Fig. 1.

#### Patient characteristics

7/46 patients (15.2%) had known malignant solid tumors whereas 20/46 patients had haematological disease (43.7%) and 4/46 (8.6%) had autoimmune disorders or were examined due to acute occurrence of atypical interstitial pneumonia. The rest of our cohort were HIV-positive (2/46, 4.3%) or had anticoagulant therapy (7/46, 15.2%), chronic lung diseases (COLD, pulmonary fibrosis) (3/46, 6.5%) and acute respiratory infection (pneumonia) (3/46, 6.5%). None of the patients except COLD-patients had pre-existing lung pathologies (e.g. related to the underlying autoimmune disorder).

#### Clinical and laboratory patient data

All patients presented with respiratory symptoms. 16/46 patients (34.7%) had neutropenia and 25/46 (54.3%) had thrombocytopenia.

### Standard of reference

According to BAL-analysis, 5/46 (10.8%) patients had RSV, 6/ 46 (13.0%) had HSV1 and 21/46 (45.6%) had PJP. Alveolar hemorrhage was diagnosed by BAL in 14/46 patients (30.6%).

Assignment of the patients to one of the three categories viral or PJP pneumonia and diffuse alveolar hemorrhage was based on microbiological data collected by BAL or by evidence of blood in the bronchial lavage. Diagnosis of herpes simplex virus pneumonia was based on the isolation of the virus by cell culture. Monolayers of human foreskin fibroblasts and vero cells were inoculated with bronchoalveolar lavage (BAL) and maintained in culture for up to 2 weeks. The virus was identified by its characteristic cytopathic effect and immunoperoxidase staining for HSV glycoprotein D.

Detection of respiratory syncytial virus (RSV) from BAL was done by real time PCR using a commercially available assay according to the instructions of the manufacturer (RealStar RSV RT-PCR Kit, altona Diagnostics GmbH, Hamburg, Germany). All patients with fresh alveolar hemorrhage had blood in the BAL.

#### CT examination protocol

All chest-CTs were obtained at end-inspiratory phase. No IV contrast medium was given. In total, 46 CT-examinations were performed. CT-examinations were performed using multidetector CT-scanner (Somatom Sensation 16/64 or Definition AS Plus, Siemens Medical Systems, Erlangen, Germany), a 250–330 mm field of view, a 512 × 512 reconstruction matrix, 120 kV, 100–150 effective mAs and a tube rotation time of 0.5 ms. In all patients a spiral acquisition was obtained from the apex to the base of the lungs. Thin-slice CT scans were reconstructed using a sharp (filter, B70f) reconstruction algorithm for visual assessment and a soft tissue kernel (filter, B31f) for CTTA. No iterative reconstruction was used. All chest-HRCTs were first analysed for the co-existence of possible complications like pleural effusions, cardiac or renal edema, and haemorrhage, bacterial (lobular or bronchopulmonal pneumonia) or viral infection in which case they had to be excluded from the final analysis.

### Imaging analysis

All scans were viewed at standard lung window (level, − 700 HU; width, 1500 HU by two independent readers (MH, CK) with 25 and 5 year experience in reading chest-CT. Image data was first analysed for the presence of lung parenchymal abnormalities (ground-glass opacity-GGO, crazy paving, air-space consolidation, reticulation, thickening of the bronchial wall and centrilobular nodules-(tree-in-bud)) including their anatomical distribution (central vs. peripheral) and predominance. Ground-glass attenuation was defined as an area of hazy increased attenuation without obscuration of underlying vascular markings. Air-space consolidation was considered present when the opacities obscured the underlying vessels. Crazy-paving was considered when parenchymal attenuation reached intermediate values between GGO and air-space consolidation enabling distinction of vascular and bronchial structures. Reticulation was defined as thickening of interlobular and intralobular septa. The presence of centrilobular nodules (tree-in-bud) as well as thickening of the bronchial walls (bronchial cuffing) was also registered. Furthermore, anatomic distribution was regarded as predominantly peripheral if abnormalities were seen mostly in the outer third of the lung vs. predominantly central if most were in the inner third of the lung. Finally, the incidence of all HRCT-imaging findings was quantified determining the predominant involvement pattern. The extent of pulmonary disease was evaluated in 3 levels of the lungs, as follows: the upper level was defined as the area above the level of the carina; the middle level, between the level of carina and the level of the inferior pulmonary veins; and the lower level, beneath the level of the inferior pulmonary veins. Each HRCT sign was separately coded as present or absent in these six areas. For each zone, a score was based on a visual estimation of the percentage of lung tissue demonstrating the CT sign (1, extent < 25%, 2: extent between 25 and 50%; 3: extent between 50 and 75%; and 4: extent > 75%). The global extent score of each sign was the sum of the 3 zonal scores [12].

HU measurements of parenchymal abnormalities were not performed as they are indirectly reflected by the mean intensity and mean average values obtained at CTTA.

**Fig. 2:**
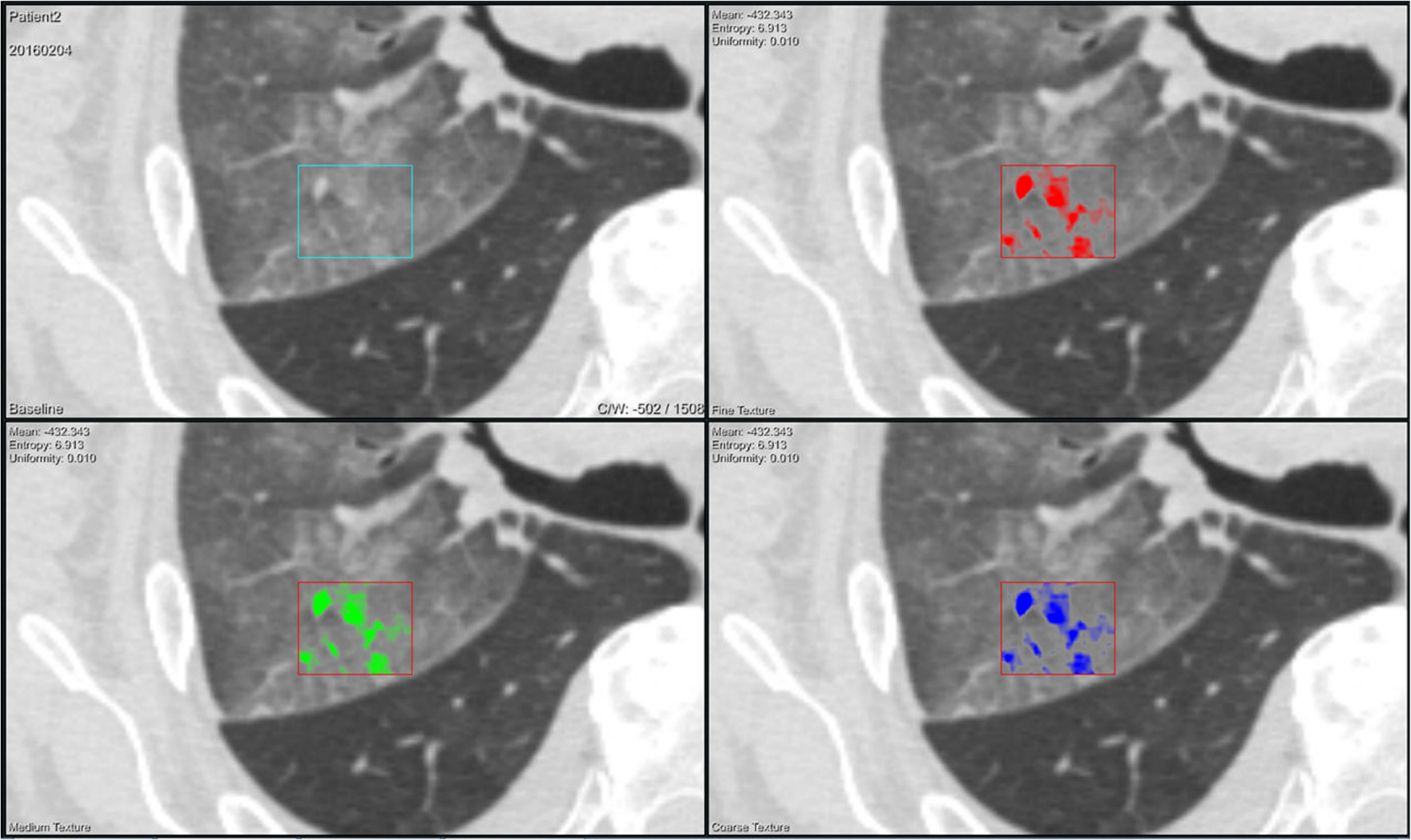
78-year-old male patient with lung hemorrhage following overdose of oral dabigatran anticoagulation therapy. Computed tomography texture analysis (CTTA) was applied on the detected haemorrhage in the right upper lobe These color-coded CTTA map display the mean intensity of the haemorrhage documented by a fine (*red*), medium (*green*), and course (*blue*) filter overlaying morphologic CT-image data

In the next step large volumes of interest (VOI) were set in the most representative areas in both lungs (if both involved) in order to perform texture analysis (Fig. 2).

### CT-texture analysis

CTTA was evaluated using a prototype software (Siemens Healthcare) based on image data sets of 1 mm slice thickness. HU units between − 600 and + 200 HU were involved [13]. The computation of each texture type for an input volume of interest (VOI) involved assigning a new value (“texture value”) to all voxel of that VOI. This technique includes an image filtration step to selectively extract features of different sizes and intensity variation, followed by texture quantification. Series of derived images at different spatial scales from fine to coarse texture within a volume of interest (VOI) drawn in lung.

The VOIs were positioned on typical disease manifestations both in the right and the left lung. A total of 82 VOIs were drawn, 41 in the left lung and 41 in the right lung. VOIs were placed only in the left or right lung in case of unilateral disease involvement. The areas were the VOIs were set was decided by the senior reader (25 years’ experience in thoracic imaging) and represented the predominantly involved lung areas and CT-features.

As we addressed a mixed of viral and fungal pneumonias as well as lung hemorrhage in early stages, filter parameter were tuned for medium texture. A computation was performed on the current voxel and its neighbourhood, and the results of that were stored as the current voxel’s texture value. This was repeated for every voxel in the VOI. All computed CTTA-parameters and their meaning are shown on Table 2.

**Table 2:** Overview of textural features with definitions subdivided into textural features of 1st and 2nd-order

1st-order textural features	
Heterogeneity	= presence of edges detected by the use of a Laplacian of Gaussian filter
Intensity	= texture intensity as the voxel value of the corresponding input image voxel
Average	= noise independent voxel intensity
Deviation	= correlates with the local range of input image voxel values
Skewness	= describes if the current neighbourhood has a centered distribution of grey values
2nd-order textural features	
Entropy of co-occurrence matrix	= entropy of the distribution of two co-occurring neighbour grey values
Number non-uniformity (NGLDM)	= the sum of squared NGLDM matrix elements divided by the sum of (unsquared) matrix elements
Entropy of NGLDM	= considers NGLDM matrix entries as random variables with an underlying statistical distribution, an image with a certain kind of regularity
Entropy of heterogeneity	= the randomness on the presence and distribution of edges
Entropie (NGLDM)	= considering NGLDM matrix entries as random variables with an underlying statistical distribution, an image with a certain kind of regularity
Contrast (NGTDM)	= correlation of grey value differences between neighbouring voxels (Difference_greyValueNeigbors_) with the range of voxels in the whole neighbourhood of the current voxel (Range_neighborhood_). The texture value for the current voxel is computed as: textureValue_currentVoxel_ = Range_neighborhood_ * Difference_greyValueNeigbors_

*Abbreviations: NGLDM* Neighbouring Grey-Level Dependence Matrix

### Statistics

All results are expressed as average with standard deviation. Statistical analysis was performed using dedicated software (IBM SPSS 22.0 (SPSS, Armonk, USA). The Kolmogorow-Smirnov test was used for the normality test including Lilliefors significance correction. For correlation Pearson’s correlation coefficient were calculated.

For comparing texture parameters, a Bonferroni correction was applied. A value of *p* < 0.0125 was considered significant. Predictive performance was assessed in the validation cohort by estimating AUC with receiver-operating-characteristic (ROC) curves. Inter-observer agreement was tested by Kendall tau correlations coefficient.

## Result

### Visual HRCT-analysis (image findings, distribution, and predominance)

Incidence of GGO, crazy-paving, air-space consolidation, centrilobular nodules/tree-in-bud, thickening of bronchial wall and reticulation is presented on Table 3. No statistical significant differences were registered for GGO (*p* = 0.703), crazy-paving (*p* = 0.0509), centrilobular nodules (*p* = 0.997) and parenchymal consolidations (*p* = 0.478) between the different subgroups.

**Table 3:** Patterns of lung involvement and predominance according to visual high-resolution CT analysis

	GGO	Crazy-paving	Centrilobular nodules/ Tree in bud	Thickening of bronchial wall	Reticulation	Air-space consolidation	Peripheral vs. central zone	Dominant pattern
Alveolar hemorrhages (A)	14/14 (100%)	9/14 (64.2%)	9/14 (64.2%)	3/14 (21.4%)	10/14 (71.4%)	11/14 (78.5%)	8/14 zentral (57.1%)	air-space consolidation (5/14, 35.7%)
PJP (B)	19/21 (90.4%)	10/21 (47.6%)	6/21 (28.5%)	4/21 (19.0%)	4/21 (19.0%)	14/21 (66.6%)	13/21 peripher (61.9%)	GGO (7/21, 33.3%)
Virus pneumonia (C)	9/11 (81.8%)	6/11 (54.5%)	7/11 (63.6%)	2/11 (18.1%)	3/11 (27.2%)	5/11 (45.4%)	10/11 peripher (90.9%)	air-space consolidation (4/11, 36.3%)

*Abbreviations: GGO* ground-glass opacity, *PJP pneumocystis jirovecii* pneumonia

The predominant pattern of involvement was air-space consolidation for alveolar hemorrhage (35.7%) and viral pneumonia (36.3%) whereas for PJP, GGO was predominant. However, differences in this respect did not reach statistical relevance (*p* = 0.245).

Statistical significant differences were registered for reticular consolidations (significant more often in alveolar haemorrhages, *p* = 0.012) and distribution pattern of peripheral and central lung pattern (*p* = 0.021). According to this data, peripheral distribution was significantly more often registered in viral pneumonia than in alveolar haemorrhages and PJP. Exact distribution is shown on Table 3.

A subpleural sparing was found in 12/21 PJP, 6/14 alveolar hemorrhages and 4/11 viral pneumonias.

The presence of crazy-paving correlated significantly with peripheral distribution in PJP patients (*p* = 0.002, r = 0.626).

ROC analysis for estimation of cut-off values based on CTTA parameters yielded no significant values for predicting of one of the three subgroups. Interobserver agreement was k = 0.579 for evaluation (*p* = 0.001).

### CTTA analysis

Discriminatory CTTA-features were found between diffuse alveolar hemorrhage and PJP consisting of differences in mean heterogeneity (*p* < 0.015) and uniformity of skewness (*p* < 0.006). Statistically significances were tabulated on Table 4. Detailed value results of CTTA-analysis were shown on Additional file 1: Table S1.

**Table 4:** Textural features comparing alveolar hemorrhages (A), PJ-pneumonias (PJP) (B) and. HSV1 = herpes simplex virus-1 pneumonias (C). A value of *p* < 0.025 was considered significant. The scale was selected by tuning the fine filter parameter (fine texture features of 4 pixels in width)

	*p* alveolar haemorrhages vs. PJ-pneumonias			*p* PJ-pneumonias vs. herpes simplex virus-1 pneumonias			*p* hemorrhages vs.herpes simplex virus-1 pneumonias		
Medium filter	Mean	Entropy	Uni-formity	Mean	Entropy	Uni-formity	Mean	Entropy	Uni-formity
Heterogeneity	0.015*	0.568	0.991	0.420	0.091	0.054	0.155	0.212	0.144
Intensity	0.485	0.969	0.502	0.260	0.130	0.097	0.671	0.210	0.096
Average	0.450	0.527	0.879	0.224	0.029	0.052	0.653	0.159	0.067
Deviation	0.046	0.065	0.128	0.036	0.033	0.061	0.803	0.382	0.173
Skewness	0.617	0.060	0.006*	0.095	0.862	0.287	0.543	0.133	0.169
Entropy (Co-occurrence Matrix)	0.432	0.722	0.592	0.133	0.089	0.085	0.533	0.120	0.183
Difference Variance (Co-occurrence Matrix)	0.584	0.621	0.601	0.858	0.098	0.095	0.469	0.345	0.113
Number non-uniformity (NGLDM)	0.583	0.699	0.385	0.722	0.034	0.047	0.410	0.036	0.143
Entropy (NGLDM)	0.043	0.886	0.530	0.261	0.143	0.076	0.610	0.119	0.087
Contrast (NGTDM)	0.635	0.836	0.287	0.219	0.040	0.095	0.526	0.123	0.248

*Abrreviations: NGLDM* Neighboring Grey-Level Dependence Matrix.

There was no difference between CT-textural features of diffuse alveolar hemorrhage and viral pneumonia or PJP and viral pneumonia.

### Correlations (medium filter)

*a)* Alveolar hemorrhages **(A).**

Crazy paving correlated significantly with mean intensity (*p* = 0.016, r = 0.630), entropy of intensity (*p* = 0.013, r = 0.644), mean average (*p* = 0.013, r = 0.642), entropy of average (*p* = 0.004, r = 0.711) and uniformity of average (*p* = 0.025, r = **−** 0.595).

*b)* PJP **(B).**

Centrilobular nodules correlated with entropy of intensity (*p* = 0.013, r = **−** 0.607) and uniformity of intensity (*p* = 0.019, r = 0.577). Furthermore the presence of centrilobular nodules correlated with entropy of average (*p* = 0.016, r = **−** 0.589) and uniformity of average (*p* = 0.018, r = 0.583).

Mean skewness correlated with the presence of centrilobular nodules (*p* = 0.014, r = − 0.598).

*c)* Virus pneumonia **(C).**

Uniformity of entropy of co-occurrence matrix correlated significantly with reticular consolidation (*p* = 0.001, r = 0.991).

Results of fine and course filter were added as Additional file 1: Table S1.

## Discussion

Discrimination between infectious and non-infectious pulmonary complications in high-risk patients (e.g. after bone marrow transplantation) is challenging and image-based diagnosis using high-resolution chest-CT is helpful, but mostly not specific enough [3, 14]. Therefore BAL is usually required despite high-risk of bleeding due frequently to accompanying thrombocytopenia [15, 16].

In this study, we have aimed at assessing the potential benefit of using a new post-processing technique based on chest-CT image data called texture analysis for differentiation between two common types of viral pneumonia (RSV and HSV1), fungal (PJP) and a non-infectious frequent pulmonary complication the alveolar hemorrhage that usually occurs in the clinical setting of immunosuppression related e.g. to malignant hematologic diseases. In order to correctly interpret our results we performed a literature data research focusing on reports that dealt with histologic changes in early stages of these pulmonary complications looking for correlations to our CTTA-features (proof of principle) [12, 14, 17]. Additionally, we evaluated all cases by visual assessment of HRCT-data considering conventional imaging findings including their distribution and predominance.

Our results are sobering in terms of discriminatory CTTA-features between these pulmonary complications showing only significant differences between PJP and alveolar hemorrhage in terms of mean heterogeneity and uniformity of skewness. Differences between PJP and alveolar hemorrhage were found also using visual assessment of the most common image findings expected in these disorders with predominance of GGO in PJP and of air-space consolidation in viral pneumonia and alveolar hemorrhage. Reticulation and centrilobular nodules were found more commonly in alveolar hemorrhage vs. PJP whereas peripheral lung parenchymal involvement was more frequent in viral pneumonias. Nonetheless, visual assessment showed great overlap of imaging findings with no statistical relevance of results. Interestingly, the second order statistics parameters reflecting the pulmonary microarchitecture including uniformity in distribution of pulmonary findings varied to a very low degree. Considering that histologic changes related to viral or fungal (PJP) infections or alveolar hemorrhage affect in a similar way the lung parenchyma at least in the early phases of the disease, CTTA-results should not differ indeed between these categories which would imply that there is little benefit if applying this new post-processing technique.

### Imaging findings

Chest-CT manifestations also significantly overlap with all six imaging features being present in all our patients. Notably, the mean intensity in alveolar hemorrhage was not increased as expected. This is presumably due to the early phase of hemorrhage where the hematocrit is still low and thus the attenuation remains in the range also expected from air-space consolidation. According to visual CT-assessment, in PJP GGO generally predominated whereas in alveolar hemorrhage air-space consolidation was more frequent which in our opinion explains differences in tissue heterogeneity and the entropy of skewness between these two pulmonary complications.

### CTTA

The potential benefit of using CT-textural features for characterization of lung parenchymal abnormalities has been already demonstrated in previous reports [10, 11, 18]. The main strength of this technique consists in its histogram-based ultrastructural tissue analysis which enables to some extent parallels macro-histology. This is particularly true in the lung which has a predictable three-dimensional texture. Behind 1st order statistical features delivering information about tissue intensity, structural heterogeneity and or uniformity, 2nd order statistics give a more profound insight into the tissue buildup analyzing the matrix (each single voxel) and deriving sensitive data with respect to their spatial distribution. In the past, numerous texture features characterizing grey-level intensity and distribution have been correlated with the tumor microarchitecture defined by histology [13, 19]. Some few reports have already addressed the issue of using CT textural analysis for characterization of lung expanding its application area [11, 20]. Park et al. found a better correlation of texture-based quantification of lung emphysema with PFT compared to density-based evaluation [10]. Cunliffe et al. applied CT textural analysis for monitoring the course of radiation-induced pneumonitis defining specific CT textural analysis features capable to accurately accomplish this task [11, 21]. However, the prerequisite for a successful performance of this technique is a histology-based differentiation between normal and abnormal lung parenchyma on the one side and also between pulmonary abnormalities to be characterized on the other side. At this point, our data confirms the potential of CT-textural features for tissue characterization, but also its limits in case of similar pathologic tissue changes as with the disorders that we have addressed in our study.

### HRCT and CT textural features correlations

The different imaging features detected by visual evaluation of HRCT-scans showed some good correlations with CT-textural features. Hence, crazy-paving in cases with alveolar hemorrhage correlated positively with mean intensity and entropy of intensity as well as with mean average and entropy and uniformity of average (noise independent voxel intensity) and negatively with the uniformity of average. This is presumably a measure for the distribution of parenchymal attenuation in this pulmonary complication.

In viral pneumonias, reticulation correlated well with the uniformity of entropy of co-occurrence matrix which represents the distribution of neighbors (voxel with a certain grey value) in a CT-morphological lattice-like attenuation pattern. Finally, the presence of centrilobular nodules in PJP correlated positively with the uniformity of intensity and average and negatively with the entropy of intensity and average. This shows how a punctiform attenuation pattern affects the relationship between neighbors in terms of regularity of intensity and probability of distribution.

### Study limitations

This study has some limitations. First, it was retrospective in character. Second, not all our patients were in the clinical setting of immunosuppression before or shortly after allogeneic bone marrow transplantation which was the hypothetical scenario for the rationale of such a project. Nonetheless, the amount of e.g. infection-related pulmonary changes is not only dependent on the immune status of affected patients but also by the virulence of the pathogen. Similarly, imaging features of alveolar hemorrhage occurring in the setting of overdosed anticoagulant drugs or due to leukemia or temporarily post-treatment thrombocytopenia are expected to exhibit similar histologic features. Third, the number of cases retrospectively included was small for a robust statistical evaluation.

Nonetheless, we consider that research on this topic should be extended and focussed on CTTA-differences between HRCT-image findings irrespective of their cause aiming at automated detection and classification of such features by using machine learning algorithms. Our report is only a proof of principle, further extended studies is necessary.

## Conclusion

In conclusion, due presumably to histologic similarities, viral PJ pneumonia and alveolar hemorrhage present with similar imaging features at chest-HRCT and also at CTTA. However, mean heterogeneity and uniformity of skewness proved helpful for discrimination between PJP and diffuse alveolar hemorrhage probably as a consequence of the more uniform infiltrative pattern of GGO in PJP. Nevertheless further extended studies are necessary.

## Additional file

Additional file 1: **Table S1.** Tetxure anaylsis features comparing PJP (B) and Virus pneumonia (C). A value of *p* < 0.025 was considered significant. The scale was selected by tuning the medium and course filter parameter (medium textures features of 6 to 10 pixels and coarse texture features of 12 pixels in width). Abrreviations: NGLDM =Neighboring Grey-Level Dependence Matrix (DOCX 38 kb)

## Abbreviations

AUC: Area under the curve
BAL: bronchial lavage
CMV: cytomegalovirus
COLD: chronic obstructive lung diesease
CT: computed tomnography
CTTA: computed tomography textural features
GGO: like ground-glass opacity
HIV: human immunodeficiency virus
HRCT: high resolution computed tomography
HSV1: herpes-simplex-1-virus
PCR: polymerase chain reaction
PJP: pneumocystis-jirovecii-pneumonia
ROC: receiver-operating-characteristic
RSV: respiratory-syncitial-virus
VOI: volume of interest
