# Supplementary material for: Potential role of CT-textural features for differentiation between viral interstitial pneumonias, pneumocystis jirovecii pneumonia and diffuse alveolar hemorrhage in early stages of disease: a proof of principle
Source: BMC Med Imaging. 2019 May 21;19:39. doi: 10.1186/s12880-019-0338-0 (PMC6530105; doi:10.1186/s12880-019-0338-0)
Supplement: Supplementary file 1 — Table S1. Tetxure anaylsis features comparing PJP (B) and Virus pneumonia (C). A value of p < 0.025 was considered significant. The scale was selected by tuning the medium and course filter parameter (medium textures features of 6 to 10 pixels and coarse texture features of 12 pixels in width). Abrreviations: NGLDM =Neighboring Grey-Level Dependence Matrix (DOCX 38 kb) [file 12880_2019_338_MOESM1_ESM.docx]

|  | Alveolar haemorraghes **(A)** | | | PJP **(B)** | | | Virus pneumonia **(C)** | | | ***p (A vs B)*** | | |
| --- | --- | --- | --- | --- | --- | --- | --- | --- | --- | --- | --- | --- |
| **Fine filter** | Mean | Entropy | Uni-formity | Mean | Entropy | Uni-formity | Mean | Entropy | Uni-formity | Mean | Entropy | Uni-formity |
| Heterogeneity | 9.2210 | 6.3838 | 0.0202 | 6.3121 | 6.2798 | 0.0188 | 9.2674 | 6.6891 | 0.0120 | *0.071* | *0.0691* | *0.828* |
| Intensity | -456.3616 | 6.2798 | 0.0266 | -471.3952 | 6.2920 | 0.0205 | -444.7660 | 6.7401 | 0.0121 | *0.485* | *0.969* | *0.473* |
| Average | -447.5799 | 5.8981 | 0.0562 | -463.2488 | 5.8199 | 0.0425 | -434.0971 | 6.5899 | 0.0143 | *0.486* | *0.856* | *0.568* |
| Deviation | 62.3952 | 5.7146 | 0.0332 | 54.4230 | 5.2771 | 0.0471 | 66.3865 | 6.0298 | 0.0203 | *0.152* | *0.136* | *0.259* |
| Skeweness | -0.1336 | 4.2648 | 0.1299 | -0.1825 | 4.5168 | 0.0956 | -0.0908 | 4.5499 | 0.1056 | *0.497* | *0.213* | *0.053* |
| Entropy (Co-occurrence Matrix) | 1.2815 | 5.5424 | 0.0905 | 1.2684 | 5.5703 | 0.0541 | 1.3864 | 6.2238 | 0.0212 | *0.914* | *0.946* | *0.331* |
| Difference Variance (Co-ocurrence Matrix) | 0.2866 | 3.7345 | 0.1487 | 0.2936 | 3.8447 | 0.1076 | 0.2627 | 4.0481 | 0.0861 | *0.872* | *0.694* | *0.270* |
| Number Nonuniformity (NGLDM) | 0.6052 | 2.8945 | 0.2098 | 0.5797 | 3.3058 | 0.1532 | 0.5691 | 3.2785 | 0.1361 | *0.443* | *0.079* | *0.133* |
| Entropie (NGLDM) | 1.0043 | 4.0212 | 0.0861 | 1.1443 | 4.2686 | 0.0788 | 1.0231 | 4.2507 | 0.0626 | *0.017* | *0.275* | *0.717* |
| Contrast (NGTDM) | 0.1987 | 5.5308 | 0.0660 | 0.2010 | 5.6595 | 0.0567 | 0.1982 | 5.9323 | 0.0323 | *0.911* | *0.705* | *0.730* |

|  | Alveolar haemorraghes **(A)** | | | PJP **(B)** | | | Virus pneumonia **(C)** | | | ***p (B vs C)*** | | |
| --- | --- | --- | --- | --- | --- | --- | --- | --- | --- | --- | --- | --- |
| **Fine filter** | Mean | Entropy | Uni-formity | Mean | Entropy | Uni-formity | Mean | Entropy | Uni-formity | Mean | Entropy | Uni-formity |
| Heterogeneity | 9.2210 | 6.3838 | 0.0202 | 6.3121 | 6.2798 | 0.0188 | 9.2674 | 6.6891 | 0.0120 | *0.204* | *0.114* | *0.093* |
| Intensity | -456.3616 | 6.2798 | 0.0266 | -471.3952 | 6.2920 | 0.0205 | -444.7660 | 6.7401 | 0.0121 | *0.260* | *0.130* | *0.097* |
| Average | -447.5799 | 5.8981 | 0.0562 | -463.2488 | 5.8199 | 0.0425 | -434.0971 | 6.5899 | 0.0143 | *0.223* | *0.067* | *0.059* |
| Deviation | 62.3952 | 5.7146 | 0.0332 | 54.4230 | 5.2771 | 0.0471 | 66.3865 | 6.0298 | 0.0203 | *0.055* | *0.018* | *0.046* |
| Skeweness | -0.1336 | 4.2648 | 0.1299 | -0.1825 | 4.5168 | 0.0956 | -0.0908 | 4.5499 | 0.1056 | *0.099* | *0.817* | *0.380* |
| Entropy (Co-occurrence Matrix) | 1.2815 | 5.5424 | 0.0905 | 1.2684 | 5.5703 | 0.0541 | 1.3864 | 6.2238 | 0.0212 | *0.280* | *0.087* | *0.128* |
| Difference Variance (Co-ocurrence Matrix) | 0.2866 | 3.7345 | 0.1487 | 0.2936 | 3.8447 | 0.1076 | 0.2627 | 4.0481 | 0.0861 | *0.449* | *0.436* | *0.158* |
| Number Nonuniformity (NGLDM) | 0.6052 | 2.8945 | 0.2098 | 0.5797 | 3.3058 | 0.1532 | 0.5691 | 3.2785 | 0.1361 | *0.707* | *0.889* | *0.308* |
| Entropie (NGLDM) | 1.0043 | 4.0212 | 0.0861 | 1.1443 | 4.2686 | 0.0788 | 1.0231 | 4.2507 | 0.0626 | *0.063* | *0.947* | *0.238* |
| Contrast (NGTDM) | 0.1987 | 5.5308 | 0.0660 | 0.2010 | 5.6595 | 0.0567 | 0.1982 | 5.9323 | 0.0323 | *0.858* | *0.318* | *0.108* |

|  | Alveolar haemorraghes **(A)** | | | PJP **(B)** | | | Virus pneumonia **(C)** | | | ***p (A vs C)*** | | |
| --- | --- | --- | --- | --- | --- | --- | --- | --- | --- | --- | --- | --- |
| **Fine filter** | Mean | Entropy | Uni-formity | Mean | Entropy | Uni-formity | Mean | Entropy | Uni-formity | Mean | Entropy | Uni-formity |
| Heterogeneity | 9.2210 | 6.3838 | 0.0202 | 6.3121 | 6.2798 | 0.0188 | 9.2674 | 6.6891 | 0.0120 | *0.98* | *0.188* | *0.177* |
| Intensity | -456.3616 | 6.2798 | 0.0266 | -471.3952 | 6.2920 | 0.0205 | -444.7660 | 6.7401 | 0.0121 | *0.671* | *0.121* | *0.096* |
| Average | -447.5799 | 5.8981 | 0.0562 | -463.2488 | 5.8199 | 0.0425 | -434.0971 | 6.5899 | 0.0143 | *0.625* | *0.137* | *0.143* |
| Deviation | 62.3952 | 5.7146 | 0.0332 | 54.4230 | 5.2771 | 0.0471 | 66.3865 | 6.0298 | 0.0203 | *0.563* | *0.194* | *0.102* |
| Skeweness | -0.1336 | 4.2648 | 0.1299 | -0.1825 | 4.5168 | 0.0956 | -0.0908 | 4.5499 | 0.1056 | *0.537* | *0.169* | *0.182* |
| Entropy (Co-occurrence Matrix) | 1.2815 | 5.5424 | 0.0905 | 1.2684 | 5.5703 | 0.0541 | 1.3864 | 6.2238 | 0.0212 | *0.409* | *0.139* | *0.158* |
| Difference Variance (Co-ocurrence Matrix) | 0.2866 | 3.7345 | 0.1487 | 0.2936 | 3.8447 | 0.1076 | 0.2627 | 4.0481 | 0.0861 | *0.533* | *0.276* | *0.083* |
| Number Nonuniformity (NGLDM) | 0.6052 | 2.8945 | 0.2098 | 0.5797 | 3.3058 | 0.1532 | 0.5691 | 3.2785 | 0.1361 | *0.281* | *0.048* | *0.045* |
| Entropie (NGLDM) | 1.0043 | 4.0212 | 0.0861 | 1.1443 | 4.2686 | 0.0788 | 1.0231 | 4.2507 | 0.0626 | *0.746* | *0.102* | *0.201* |
| Contrast (NGTDM) | 0.1987 | 5.5308 | 0.0660 | 0.2010 | 5.6595 | 0.0567 | 0.1982 | 5.9323 | 0.0323 | *0.977* | *0.192* | *0.167* |

|  | Alveolar haemorraghes **(A)** | | | PJP **(B)** | | | Virus pneumonia **(C)** | | | ***p (A vs B)*** | | |
| --- | --- | --- | --- | --- | --- | --- | --- | --- | --- | --- | --- | --- |
| **Coarse filter** | Mean | Entropy | Uni-formity | Mean | Entropy | Uni-formity | Mean | Entropy | Uni-formity | Mean | Entropy | Uni-formity |
| Heterogeneity | 0.2798 | 6.5268 | 0.0220 | 0.1934 | 6.2100 | 0.0241 | 0.05674 | 6.8490 | 0.0109 | *0.752* | *0.409* | *0.838* |
| Intensity | -456.3616 | 6.2798 | 0.0266 | -471.3952 | 6.2920 | 0.0205 | -444.7660 | 6.7401 | 0.0121 | *0.485* | *0.969* | *0.502* |
| Average | -445.7789 | 5.7250 | 0.0759 | -463.3417 | 5.3929 | 0.0672 | -434.1856 | 6.1621 | 0.0246 | *0.434* | *0.480* | *0.798* |
| Deviation | 75.6017 | 5.6638 | 0.0332 | 57.8251 | 5.0192 | 0.0655 | 74.9925 | 5.6927 | 0.0286 | *0.019** | *0.031* | *0.040* |
| Skeweness | -0.1033 | 3.6421 | 0.2049 | -0.1867 | 4.1183 | 0.1343 | -0.0951 | 3.8844 | 0.1784 | *0.560* | *0.052* | *0.011** |
| Entropy (Co-occurrence Matrix) | 1.5150 | 6.1875 | 0.0684 | 1.3177 | 5.8736 | 0.0574 | 1.5878 | 6.6194 | 0.0172 | *0.253* | *0.491* | *0.783* |
| Difference Variance (Co-ocurrence Matrix) | 0.3129 | 5.0946 | 0.0865 | 0.2628 | 4.7537 | 0.0801 | 0.2731 | 5.4222 | 0.0371 | *0.305* | *0.356* | *0.868* |
| Number Nonuniformity (NGLDM) | 0.4245 | 6.1944 | 0.0386 | 0.4113 | 6.1219 | 0.0319 | 0.4074 | 6.6580 | 0.01276 | *0.632* | *0.834* | *0.753* |
| Entropie (NGLDM) | 1.6522 | 6.4912 | 0.0336 | 1.8277 | 6.2866 | 0.0292 | 1.7292 | 6.8639 | 0.0106 | *0.077* | *0.561* | *0.836* |
| Contrast (NGTDM) | 0.1378 | 6.0517 | 0.0765 | 0.1230 | 5.9273 | 0.0501 | 0.1364 | 6.6060 | 0.0148 | *0.243* | *0.783* | *0.512* |

|  | Alveolar haemorraghes **(A)** | | | PJP **(B)** | | | Virus pneumonia **(C)** | | | ***p (B vs C)*** | | |
| --- | --- | --- | --- | --- | --- | --- | --- | --- | --- | --- | --- | --- |
| **Coarse filter** | Mean | Entropy | Uni-formity | Mean | Entropy | Uni-formity | Mean | Entropy | Uni-formity | Mean | Entropy | Uni-formity |
| Heterogeneity | 0.2798 | 6.5268 | 0.0220 | 0.1934 | 6.2100 | 0.0241 | 0.05674 | 6.8490 | 0.0109 | *0.390* | *0.028* | *0.078* |
| Intensity | -456.3616 | 6.2798 | 0.0266 | -471.3952 | 6.2920 | 0.0205 | -444.7660 | 6.7401 | 0.0121 | *0.260* | *0.130* | *0.097* |
| Average | -445.7789 | 5.7250 | 0.0759 | -463.3417 | 5.3929 | 0.0672 | -434.1856 | 6.1621 | 0.0246 | *0.232* | *0.124* | *0.058* |
| Deviation | 75.6017 | 5.6638 | 0.0332 | 57.8251 | 5.0192 | 0.0655 | 74.9925 | 5.6927 | 0.0286 | *0.039* | *0.068* | *0.059* |
| Skeweness | -0.1033 | 3.6421 | 0.2049 | -0.1867 | 4.1183 | 0.1343 | -0.0951 | 3.8844 | 0.1784 | *0.182* | *0.291* | *0.110* |
| Entropy (Co-occurrence Matrix) | 1.5150 | 6.1875 | 0.0684 | 1.3177 | 5.8736 | 0.0574 | 1.5878 | 6.6194 | 0.0172 | *0.129* | *0.051* | *0.097* |
| Difference Variance (Co-ocurrence Matrix) | 0.3129 | 5.0946 | 0.0865 | 0.2628 | 4.7537 | 0.0801 | 0.2731 | 5.4222 | 0.0371 | *0.776* | *0.044* | *0.068* |
| Number Nonuniformity (NGLDM) | 0.4245 | 6.1944 | 0.0386 | 0.4113 | 6.1219 | 0.0319 | 0.4074 | 6.6580 | 0.01276 | *0.896* | *0.128* | *0.090* |
| Entropie (NGLDM) | 1.6522 | 6.4912 | 0.0336 | 1.8277 | 6.2866 | 0.0292 | 1.7292 | 6.8639 | 0.0106 | *0.253* | *0.119* | *0.129* |
| Contrast (NGTDM) | 0.1378 | 6.0517 | 0.0765 | 0.1230 | 5.9273 | 0.0501 | 0.1364 | 6.6060 | 0.0148 | *0.397* | *0.104* | *0.095* |

|  | Alveolar haemorraghes **(A)** | | | PJP **(B)** | | | Virus pneumonia **(C)** | | | ***p (A vs C)*** | | |
| --- | --- | --- | --- | --- | --- | --- | --- | --- | --- | --- | --- | --- |
| **Coarse filter** | Mean | Entropy | Uni-formity | Mean | Entropy | Uni-formity | Mean | Entropy | Uni-formity | Mean | Entropy | Uni-formity |
| Heterogeneity | 0.2798 | 6.5268 | 0.0220 | 0.1934 | 6.2100 | 0.0241 | 0.05674 | 6.8490 | 0.0109 | *0.424* | *0.346* | *0.216* |
| Intensity | -456.3616 | 6.2798 | 0.0266 | -471.3952 | 6.2920 | 0.0205 | -444.7660 | 6.7401 | 0.0121 | *0.671* | *0.121* | *0.096* |
| Average | -445.7789 | 5.7250 | 0.0759 | -463.3417 | 5.3929 | 0.0672 | -434.1856 | 6.1621 | 0.0246 | *0.677* | *0.326* | *0.108* |
| Deviation | 75.6017 | 5.6638 | 0.0332 | 57.8251 | 5.0192 | 0.0655 | 74.9925 | 5.6927 | 0.0286 | *0.945* | *0.909* | *0.622* |
| Skeweness | -0.1033 | 3.6421 | 0.2049 | -0.1867 | 4.1183 | 0.1343 | -0.0951 | 3.8844 | 0,1784 | *0.954* | *0.406* | *0.461* |
| Entropy (Co-occurrence Matrix) | 1.5150 | 6.1875 | 0.0684 | 1.3177 | 5.8736 | 0.0574 | 1.5878 | 6.6194 | 0.0172 | *0.701* | *0.285* | *0.171* |
| Difference Variance (Co-ocurrence Matrix) | 0.3129 | 5.0946 | 0.0865 | 0.2628 | 4.7537 | 0.0801 | 0.2731 | 5.4222 | 0.0371 | *0.410* | *0.358* | *0.176* |
| Number Nonuniformity (NGLDM) | 0.4245 | 6.1944 | 0.0386 | 0.4113 | 6.1219 | 0.0319 | 0.4074 | 6.6580 | 0.01276 | *0.593* | *0.113* | *0.203* |
| Entropie (NGLDM) | 1.6522 | 6.4912 | 0.0336 | 1.8277 | 6.2866 | 0.0292 | 1.7292 | 6.8639 | 0.0106 | *0.904* | *0.233* | *0.198* |
| Contrast (NGTDM) | 0.1378 | 6.0517 | 0.0765 | 0.1230 | 5.9273 | 0.0501 | 0.1364 | 6.6060 | 0.0148 | *0.482* | *0.197* | *0.252* |

|  | Alveolar hemorrhages **(A)** | | | PJP **(B)** | | | Virus pneumonia **(C)** | | | ***p (A vs B)*** | | |
| --- | --- | --- | --- | --- | --- | --- | --- | --- | --- | --- | --- | --- |
| **Medium filter** | Mean | Entropy | Uni-formity | Mean | Entropy | Uni-formity | Mean | Entropy | Uni-formity | Mean | Entropy | Uni-formity |
| Heterogeneity | 0.8507 | 6.4024 | 0.0209 | -0.1083 | 6.2434 | 0.0208 | 0.0567 | 6.8490 | 0.0109 | *0.015** | *0.568* | *0.991* |
| Intensity | -456.3616 | 6.2798 | 0.0266 | -471.3952 | 6.2920 | 0.0205 | -444.7660 | 6.7401 | 0.0121 | *0.485* | *0.969* | *0.502* |
| Average | -446.7270 | 5.8383 | 0.0608 | -463.6567 | 5.5405 | 0.0651 | -434.2412 | 6.4193 | 0.0171 | *0.450* | *0.527* | *0.879* |
| Deviation | 69.5354 | 5.6604 | 0.0359 | 55.8042 | 5.0809 | 0.0660 | 71.5680 | 5.8718 | 0.0220 | *0.046* | *0.065* | *0.128* |
| Skewness | -0.1468 | 3.853 | 0.1716 | -0.1968 | 4.2600 | 0.1147 | -0.0884 | 4.2363 | 0.1354 | *0.617* | *0.060* | *0.006** |
| Entropy (Co-occurrence Matrix) | 1.4293 | 6.0021 | 0.0780 | 1.3082 | 5.8368 | 0.0572 | 1.5310 | 6.6224 | 0.0151 | *0.432* | *0.722* | *0.592* |
| Difference Variance (Co-occurrence Matrix) | 0.3014 | 4.5834 | 0.1072 | 0.2759 | 4.4117 | 0.0872 | 0.2700 | 4.8959 | 0.0495 | *0.584* | *0.621* | *0.601* |
| Number non-uniformity (NGLDM) | 0.4922 | 5.5954 | 0.0481 | 0.4763 | 5.7054 | 0.0307 | 0.4662 | 6.1641 | 0.0188 | *0.583* | *0.699* | *0.385* |
| Entropy (NGLDM) | 1.4053 | 6.3262 | 0.0345 | 1.5727 | 6.2815 | 0.0218 | 1.4585 | 6.7393 | 0.0115 | *0.043* | *0.886* | *0.530* |
| Contrast (NGTDM) | 0.1606 | 5.9375 | 0.0843 | 0.1540 | 6.0276 | 0.0414 | 0.1674 | 6.5778 | 0.0177 | *0.635* | *0.836* | *0.287* |
|  | Alveolar hemorrhages **(A)** | | | PJP **(B)** | | | Virus pneumonia **(C)** | | | ***p (B vs C)*** | | |
| **Medium filter** | Mean | Entropy | Uni-formity | Mean | Entropy | Uni-formity | Mean | Entropy | Uni-formity | Mean | Entropy | Uni-formity |
| Heterogeneity | 0.8507 | 6.4024 | 0.0209 | -0.1083 | 6.2434 | 0.0208 | 0.0567 | 6.8490 | 0.0109 | *0.420* | *0.091* | *0.054* |
| Intensity | -456.3616 | 6.2798 | 0.0266 | -471.3952 | 6.2920 | 0.0205 | -444.7660 | 6.7401 | 0.0121 | *0.260* | *0.130* | *0.097* |
| Average | -446.7270 | 5.8383 | 0.0608 | -463.6567 | 5.5405 | 0.0651 | -434.2412 | 6.4193 | 0.0171 | *0.224* | *0.029* | *0.052* |
| Deviation | 69.5354 | 5.6604 | 0.0359 | 55.8042 | 5.0809 | 0.066 | 71.5680 | 5.8718 | 0.0022 | *0.036* | *0.033* | *0.061* |
| Skewness | -0.1468 | 3.853 | 0.1716 | -0.1968 | 4.2060 | 0.1147 | -0.0884 | 4.2363 | 0.1354 | *0.095* | *0.862* | *0.287* |
| Entropy (Co-occurrence Matrix) | 1.4293 | 6.0021 | 0.0078 | 1.3082 | 5.8368 | 0.0572 | 1.5310 | 6.6224 | 0.0151 | *0.133* | *0.089* | *0.085* |
| Difference Variance (Co-occurrence Matrix) | 0.3014 | 4.5834 | 0.1072 | 0.2759 | 4.4117 | 0.0872 | 0.2700 | 4.8959 | 0.0495 | *0.858* | *0.098* | *0.095* |
| Number non-uniformity (NGLDM) | 0.4922 | 5.5954 | 0.0481 | 0.4763 | 5.7054 | 0.0307 | 0.4662 | 6.1641 | 0.0188 | *0.722* | *0.034* | *0.047* |
| Entropy (NGLDM) | 1.4053 | 6.3262 | 0.0345 | 1.5727 | 6.2815 | 0.0218 | 1.4585 | 6.7393 | 0.0115 | *0.261* | *0.143* | *0.076* |
| Contrast (NGTDM) | 0.1606 | 5.9375 | 0.0843 | 0.1540 | 6.0276 | 0.0414 | 0.1674 | 6.5778 | 0.0177 | *0.219* | *0.040* | *0.095* |

|  | Alveolar hemorrhages **(A)** | | | PJP **(B)** | | | Virus pneumonia **(C)** | | | ***p (A vs C)*** | | |
| --- | --- | --- | --- | --- | --- | --- | --- | --- | --- | --- | --- | --- |
| **Medium filter** | Mean | Entropy | Uni-formity | Mean | Entropy | Uni-formity | Mean | Entropy | Uni-formity | Mean | Entropy | Uni-formity |
| Heterogeneity | 0.8507 | 6.4024 | 0.0209 | -0.1083 | 6.2434 | 0.0208 | 0.0567 | 6.8490 | 0.0109 | *0.155* | *0.212* | *0.144* |
| Intensity | -456.3616 | 6.2798 | 0.0266 | -471.3952 | 6.2920 | 0.0205 | -444.7660 | 6.7401 | 0.0121 | *0.671* | *0.210* | *0.096* |
| Average | -446.7270 | 5.8383 | 0.0608 | -463.6567 | 5.5405 | 0.0651 | -434.2412 | 6.4193 | 0.0171 | *0.653* | *0.159* | *0.067* |
| Deviation | 69.5354 | 5.6604 | 0.0359 | 55.8042 | 5.0809 | 0.0660 | 71.5680 | 5.8718 | 0.0220 | *0.803* | *0.382* | *0.173* |
| skewness | -0.1468 | 3.8530 | 0.1716 | -0.1968 | 4.2600 | 0.1147 | -0.0884 | 4.2363 | 0.1354 | *0.543* | *0.133* | *0.169* |
| Entropy (Co-occurrence Matrix) | 1.4293 | 6.0021 | 0.0780 | 1.3082 | 5.8368 | 0.0572 | 1.5310 | 6.6224 | 0.0151 | *0.533* | *0.120* | *0.183* |
| Difference Variance (Co-occurrence Matrix) | 0.3014 | 4.5834 | 0.1072 | 0.2759 | 4.4117 | 0.0872 | 0.2700 | 4.8959 | 0.0495 | *0.469* | *0.345* | *0.113* |
| Number non-uniformity (NGLDM) | 0.4922 | 5.5954 | 0.0481 | 0.4763 | 5.7054 | 0.0307 | 0.4662 | 6.1641 | 0.0188 | *0.410* | *0.036* | *0.143* |
| Entropy(NGLDM) | 1.4053 | 6.3262 | 0.0345 | 1.5727 | 6.2815 | 0.0218 | 1.4585 | 6.7393 | 0.0115 | *0.610* | *0.119* | *0.087* |
| Contrast (NGTDM) | 0.1606 | 5.9375 | 0.0843 | 0.1540 | 6.0276 | 0.0414 | 0.1674 | 6.5778 | 0.0177 | *0.526* | *0.123* | *0.248* |

Additional file 1: **Table S1.** Tetxure anaylsis features comparing PJP (B) and Virus pneumonia (C ). A value of p< 0.025 was considered significant.

The scale was selected by tuning the medium and course filter parameter (medium textures features of 6 to 10 pixels and coarse texture features of 12 pixels in width).

Abrreviations: NGLDM =Neighboring Grey-Level Dependence Matrix
